# The Spine Lengthens During Walking in Healthy Participants, with Age-Related Changes in Kinematic Parameters

**DOI:** 10.3390/jcm14010209

**Published:** 2025-01-02

**Authors:** Carlo Albino Frigo, Alessandra Favata, Federica Camuncoli, Veronica Farinelli, Carlotte Kiekens, Calogero Malfitano, Chiara Palmisano, Stefano Negrini

**Affiliations:** 1 Department of Electronics, Information and Bioengineering, Politecnico di Milano, 20133 Milan, Italy; carlo.frigo@polimi.it; 2 Biomechanical Engineering Lab, Department of Mechanical Engineering and Research Centre for Biomedical Engineering, Universitat Politècnica de Catalunya, 08028 Barcelona, Spain; alessandra.favata@mail.polimi.it; 3 Department of Clinical and Experimental Medicine, Università di Pisa, 50126 Pisa, Italy; federica.camuncoli@polimi.it; 4 Human Physiology Section of the Department of Pathophysiology and Transplantation, University of Milan, 20122 Milan, Italy; veronica.farinelli@unimi.it; 5 IRCCS Istituto Ortopedico Galeazzi, 20157 Milan, Italy; carlotte.kiekens@isico.it; 6 Department of Biomedical Sciences for Health, University of Milan, 20133 Milan, Italy; 7 Azienda di Servizi alla Persona Istituti Milanesi Martinitt e Stelline e Pio Albergo Trivulzio, 20146 Milan, Italy; 8 Department of Neurology, University Hospital of Würzburg, 97080 Würzburg, Germany; chiara.palmisano@polimi.it; 9 Department of Biomedical, Surgical and Dental Sciences, University of Milan, 20122 Milan, Italy

**Keywords:** spine, aging, gait analysis, biomechanical phenomena, range of motion

## Abstract

**Background:** While the importance of the upper and lower limbs in locomotion is well understood, the kinematics of the trunk during walking remains largely unexplored. Two decades ago, a casual observation was reported indicating spine lengthening in a small sample of mostly children during walking, but this observation was never replicated. Objectives: This study aims to verify the preliminary observation that spine lengthening occurs during walking and to explore changes in spine kinematics across three different age groups. **Methods:** A convenience sample of 45 healthy participants was divided into three groups of 15 individuals each: children (ages 5 to 13), young adults (ages 18 to 30), and older adults (ages 50 to 70). The spinal length, defined as the distance between C7 and the coccyx, and other kinematic parameters were analyzed using a motion analysis system while participants stood and walked standing and walking at their natural cadence. **Results:** In all groups, the length of the spine increased while walking compared to standing. This change was primarily due to a reduction in the inferior spinal angle, which is associated with lumbar lordosis, rather than a change in the superior spinal angle, which is related to thoracic kyphosis. The average change in spinal length during the walking cycle was approximately 7% in children, while it was only about 1% in adults. We also found a reduction in the range of motion for almost all the variables in adults. **Conclusions:** The increase in the spinal length during walking is related to a reduction in the physiological spine curve. This occurs due to muscle contractions which are needed to stabilize the locomotor system. As people age, the reduction in spinal length changes is associated with decreased spinal mobility and to a natural tendency toward anterior trunk flexion.

## 1. Introduction

Knowledge about spine neurophysiological control is still quite scant [1,2]. While the movement of the limbs during walking is widely studied, the behavior of the trunk remains less understood [3,4,5]. Twenty years ago, a preliminary exploratory study was presented on the change in spinal length during walking compared to standing [6]. The investigation involved 20 adolescents (10 males and 10 females, 10 to 12 years old), 11 adults (8 males, 3 females, 20–30 years old), and 1 older person (male, 67 years old). The most surprising and interesting result was that while walking, the distance between the C7 spinal process and the coccyx, which we referred to as “spinal length”, increased significantly compared to the distance measured in standing conditions. Looking into the factors contributing to this increase in spinal length, the reduction in spinal lordosis appeared as the main causal factor. Furthermore, the significant reduction in spinal length changes between teenagers and adults could demonstrate decreased spinal mobility, as previously described in other works [7,8]. Surprisingly, to the best of our knowledge, no similar observations have been reported in the literature till now.

In a previous work [9], a movement analysis protocol was developed to provide a description of the relative movements of spine segments, shoulder girdle, and pelvis during walking, but the study involved only eighteen young, healthy women. Other interesting publications present kinematic data about spine angles in different age populations [10,11] and recognize the need for a deeper knowledge of spine kinematics, also given its implication in conditioning gait and posture in several clinical conditions, such as Parkinson’s disease, low back pain, and scoliosis [12,13,14,15].

Based on our previous experience, this study aims to confirm that spinal length increases during walking and that this change reduces with aging. Additionally, we aim to investigate whether and how these changes may relate to other kinematic variables and if they are consistent across different age groups. We found it compelling to investigate the feasibility of capturing dynamic spinal changes within a movement analysis setting. This approach holds potential for enhancing clinical assessments of spinal pathologies by providing a straightforward method for estimating spinal kinematic patterns.

## 2. Materials and Methods

### 2.1. Study Design

We conducted an observational study on a healthy population. The experimental procedure was carried out according to the standards of the Declaration of Helsinki [16]. The Ethical Committee “Comitato Etico di Ateneo dell’Università degli Studi di Milano” approved the study and the written consent procedure on 15 February 2016 (counsel 5/16).

All the participants/parents/guardians signed a written informed consent form before participation.

### 2.2. Participants

We enrolled a convenience sample of 45 healthy individuals subdivided into three groups of 15 participants each: children aged 5 to 13 (C), young adults aged 18 to 30 (YA), and older adults aged 50 to 70 (OA). They had to define themselves as “healthy”. We excluded from the analysis people with spinal diseases, major orthopedic surgeries, and neurological disorders. We also excluded people with back pain in the last 30 days preceding the experiment.

### 2.3. Experimental Setup

The participants were analyzed while walking barefoot and wearing sports clothes. They were asked first to perform a standing trial while remaining still and relaxed, with their eyes open and arms at their sides for at least 30 s, without making any voluntary movements. After the standing trial, participants were instructed to walk along a linear eight-meter-long pathway at a self-selected speed. The task was repeated three to eight times according to the participants’ compliance. Participants were allowed to rest between trials.

### 2.4. Kinematic Recordings

**Figure 1:**
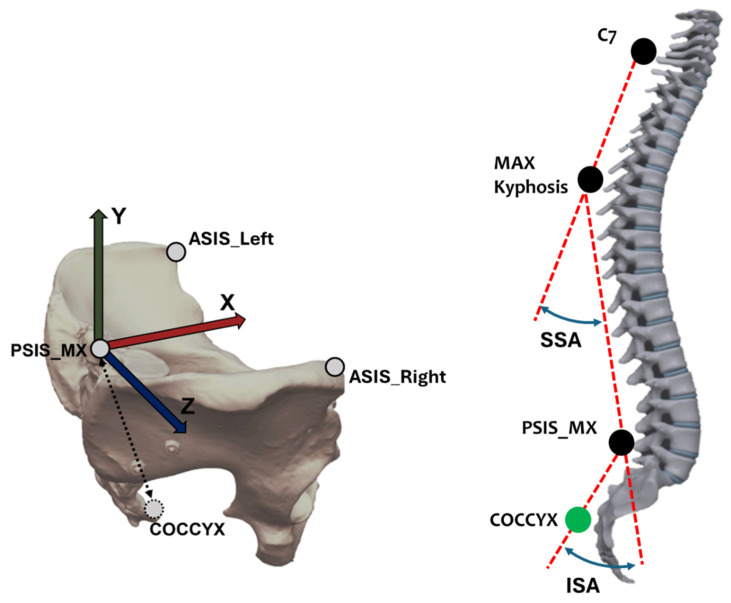
**Left**: t = The pelvis reference system and the reconstructed coccyx position. Markers positioned on ASIS_Right and ASIS_Left and the midpoint between posterior superior iliac spines (PSIS_MX) were used to define a local reference system of the pelvis: the origin in the PSIS_MX, and *X* axis oriented forward, *Y* axis oriented upwards, and *Z* axis oriented laterally to the right. **Right**: Schematization of the spine curves. SSA is the Superior Spinal Angle (associated with dorsal kyphosis), and ISA is the Inferior Spinal angle (associated with lumbar lordosis). The green circle on the coccyx indicates a virtual point, mathematically reconstructed; the black circles represent markers applied to the subject.

Kinematic data were collected utilizing a motion analysis system (Smart-E, BTS, Italy) with 6 cameras (sampling frequency of 60 Hz), using a full-body marker set [9]. Measures regarding the spine were obtained from markers positioned on the seventh cervical vertebral process (C7), the point of maximum kyphosis, and the midpoint between the two posterior superior iliac spines (PSIS_MX in Figure 1). To identify the lower extremity of the spine, we reconstructed the position of the coccyx in the local reference system of the pelvis according to previous radiographic studies [17,18,19]. The origin of the pelvic reference system was assumed to be the PSIS_MX point. The three cartesian axes were defined as follows: the anteroposterior (*X*) axis joining the PSIS_MX and the midpoint between the two anterior superior iliac spines (ASIS), pointing forward; the vertical (*Y*) axis obtained as the perpendicular to the plane which includes the two ASIS and the PSIS_MX, pointing cranially; and the mediolateral (*Z*) axis defined as the axis perpendicular to both the X and Y axes pointing right (Figure 1, left).

The coordinates of the coccyx in the local pelvis reference system were estimated as previously described in Palmisano et al., 2024 [20]:

For children: [xc = −0.05 m, yc = −0.08 m, zc = 0 m].

For adults: [xc = −0.06 m, yc = −0.09 m, zc = 0 m].

The following ‘spinal variables’ were computed to describe the spine mobility:

- Spinal length (SL), defined as the distance between C7 and the coccyx;
- Superior spinal angle (SSA), defined as the angle between the two spinal tracts, respectively, above and below the point of maximum kyphosis (see Figure 1, right) projected to the sagittal plane of the pelvis; this is a large approximation of the kyphosis angle that is generally calculated between T1-T6-T12;
- Inferior spinal angle (ISA), defined as the angle between the two spinal segments, respectively, above and below the midpoint of the PSIS (see Figure 1, right), projected onto the sagittal plane of the pelvis; this is a very large approximation of the lordosis angle that is generally calculated between T12-L3-S1;
- Regarding the pelvis, tilt in the sagittal plane, horizontal rotation, and obliquity in the frontal plane were identified according to the rotations of the pelvis local reference frame in relation to the laboratory reference frame;
- Trunk inclination was computed as the angle between the vertical axis of the laboratory and the vector connecting C7 and the midpoint between the PSIS, and projected to the sagittal (Forward Trunk Tilt) and frontal (Lateral Trunk Tilt) planes of the laboratory.

**Figure 2:**
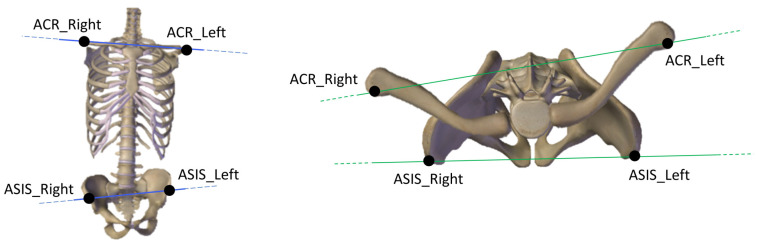
**Left**: Shoulder Girdle Inclination. Markers positioned on the left and right acromion processes (ACR), and the two anterior superior iliac spines (ASIS) projected to the frontal plane. **Right**: Shoulder Girdle Rotation. Markers positioned on the left and right ACR, and the two ASIS projected to the pelvis.

Finally, we computed the shoulder orientation angles in relation to the pelvis as the angles between the vectors connecting the two markers placed on the left and right acromion processes and the vector connecting the two ASIS projected to the frontal and horizontal planes of the pelvis. We named these angles “Shoulder Girdle Rotation” and “Shoulder Girdle Inclination,” respectively (Figure 2).

### 2.5. Data Analysis

Data were low-pass filtered with a Butterworth 3rd-order filter with a 10 Hz cut-off frequency [21]. For the standing task, the average of each variable was computed over the whole trial duration. For the walking trials, we identified left and right gait cycles using the marker placed on the heels, and each variable was averaged along the stride cycle. After resampling each variable, the ensemble average was calculated to obtain 100 data points in each gait cycle using cubic spline interpolation. Then, for each data point, each variable was averaged across all identified gait cycles, and the standard deviation was computed.

The SL variation between walking and standing conditions was expressed as a percentage of the SL measured in the standing condition:

$$SL\;variation=\frac{{(SL}_{WALKING}-{SL}_{STANDING})}{\left({SL}_{STANDING}\right)}*100\;[\%]$$

All computations were performed using ad hoc algorithms in Matlab^®^ (R2021b, The Mathworks, Natick, MA, USA).

### 2.6. Statistics

For each parameter and condition, we removed the outliers from the analysis using the Jackknife distance, i.e., the “leave one out” technique [22]. Then, all the variables were tested for normality distribution by the Anderson–Darling test. For the comparison between conditions (i.e., walking vs. standing) within each group, we applied a paired-sample *t*-test or a Wilcoxon signed rank test according to the data distribution. For the comparison among groups within each condition, we applied a Kruskal–Wallis test or ANOVA, followed by Dunn–Sidak or Tukey–Kramer post hoc analysis, as appropriate. The level of significance was set to 0.05 with Bonferroni adjustment for multiple comparisons.

## 3. Results

Table 1 reports the demographic and anthropometric data of the 45 enrolled participants. As expected, we found statistically significant differences for age, height, weight, and body mass index (BMI) between children and both young adults and older adults, and for age and BMI between young adults and older adults.

### 3.1. Comparison Within Groups Between Walking and Standing

**Figure 3:**
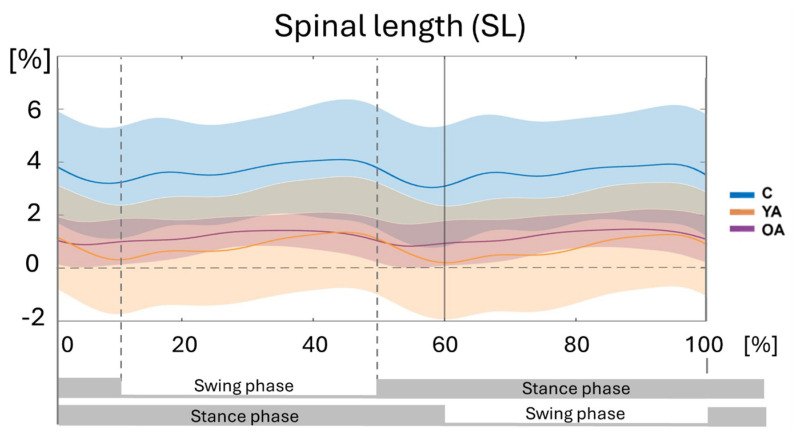
Time course of the spinal length (SL) along the gait cycle expressed in percentage of the standing spinal length (0 reference line, in the figure). Children: C (blue line); young adults: YA (orange line); and older adults: OA (purple line). Shadowed areas represent the standard deviations of the groups.

In all groups, the spinal length (SL) increased during walking in relation to the standing condition (See Figure 3). The average SL over the walking cycle (median and range for each group are reported in Table 2) is higher in children (about 3 cm, 7.2% of the standing SL) and lower in adults (about 0.72 cm, 1.3% in YA, and 0.63 cm, 1.1% in OA), although the difference is not significant (*p* < 0.05) in young adults. In parallel, the median value of the average of the Inferior Spinal Angle along the gait cycle decreased by almost 8° in children and 2–3° in both adult groups, while Forward Trunk Tilt increased by 2–3° in all groups (Table 2). The median value of the average superior spine angle did not show statistically significant variations between the walking and standing conditions in any group. During walking, the children showed a decreased median Pelvic Tilt (−5°) and Obliquity (−1°). Girdle Rotation increased by 5° in children, and Girdle Inclination decreased by 3° in older adults. Interestingly, the decreases in Inferior Spine Angle and Pelvic Tilt were significant in children (8.3° and 4.7°, respectively) and smaller or absent in adults (in both cases not significant) (Table 2).

### 3.2. Effect of Age on the Spine Parameters in Standing and Walking

As shown in Table 2, no statistical difference appeared when comparing the two adult groups during the standing condition for any of the variables considered. Significant differences were observed between young adults and children and between older adults and children. Looking at the most interesting ones, it appears that aging was characterized by an increased median Forward Trunk Tilt (increase of 1.4° in YA and 2.5° in OA), decreased inferior spinal angle (−5.0° in YA and −1.5 ° in OA), and decreased Pelvic Tilt (−6.1° in YA and −3.5° in OA). The differences between children and adults found in standing were also confirmed during gait, except for the Forward Trunk Tilt, Inferior Spine Angle, Pelvic Tilt, Pelvic Obliquity, and Girdles Rotation.

**Figure 4:**
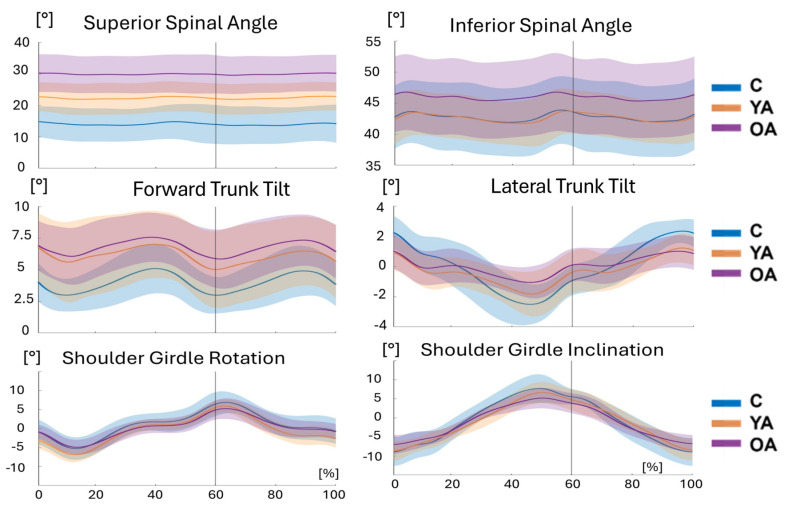
Time course of the main spinal variables along the gait cycle for the three groups analyzed. The average curve (solid line) and the standard deviation (shadowed area) are represented for each group. Children (C, blue line), young adults (YA, orange line), and older adults (OA, purple line).

Figure 4 shows the patterns of the main spinal angles during the gait cycle in the three groups. While the Superior Spinal Angle did not change much during the gait cycle, this was not true for all the other spinal angles.

When comparing the range of motion (ROM) across groups during walking (Table 3), the larger differences concerned the Pelvic Rotation (7.7° between OA and C and 3.9° between YA and C) and Lateral Trunk Tilt (2.3° between OA and C and 2.0° between YA and C). The ROM of all remaining parameters, except for the Shoulder Girdle Rotation, also showed a clear reduction trend with age. The difference was almost always statistically significant only for the comparison between children and older adults, with few exceptions (Table 3).

## 4. Discussion

This study has confirmed the previous observation that, compared to the standing upright condition, the spine length increases during walking and remains above the standing value throughout the gait cycle [9]. The differences observed were statistically significant in children and older adults, with average spinal length variations of 7.2% (nearly 3 cm) and 1.2% (0.6 cm). In young adults, the variation was 1.3% (0.7 cm). These data are consistent with our preliminary work, where the increase was 5.1% in teenagers, 1.2% in adults, and 1.3% in the elderly subject [6]. The key finding explaining this spinal lengthening is a decrease in the Inferior Spinal Angle (associated with physiological spine lordosis) and not in the Superior Spinal Angle (related to kyphosis). Interestingly, spinal lengthening was consistent at all the ages we evaluated, but its magnitude decreased with age. An increase in rigidity could explain this result. Our study’s findings align with previous knowledge on the aging of the spine, which is characterized not only by a reduction in length but also by an increase in rigidity [8,23,24,25].

When comparing the three groups, we also have documented a reduction in the range of motion during walking with age for nearly all studied variables (not statistically significant for Pelvic Tilt), and this finding, although not surprising, adds to the body of knowledge on spinal biomechanics.

The question now is what could be the functional reason for spine lengthening during gait? From a biomechanical point of view, during walking, (1) the spine must transfer forces from the head, trunk, and upper limbs (the so-called HAT) to the pelvis [26], and (2) the spine must be stabilized in the frontal and sagittal planes [16]. A contraction of the Erector Spinae Muscles is required for this purpose [27], and in theory, it should produce a shortening of the spine, particularly because of the physiological spine curves, which would increase. If we consider lumbar lordosis, the consequence of an increase in the curvature would correspond to an increased lever arm of the vertical load in relation to the vertebral bodies and, as a consequence, an increase in the load on muscles that oppose the spine curvature on spine ligaments and intervertebral disks. As demonstrated in our study, lumbar lordosis is reduced during walking, thus avoiding these detrimental effects.

However, reducing the lumbar lordosis would entail an increased forward leaning of the trunk. Here, an interesting difference in the behavior of children and adults appears: in our child population, the trunk inclination in the sagittal plane increased very little (from 1.35° to 3.61° on average) while the forward pelvis tilt decreased by almost 5° (from 12.33° to 7.66° on average). In young and old adults, the Pelvic Tilt did not change significantly, while the Forward Trunk Tilt increased by approximately 3.3° in both young and old adults. These data clearly show how spine mobility structurally changes with age: in children, the greater mobility allows for reducing lumbar lordosis in walking by reducing pelvis anteversion and keeping the column almost vertical; in adults, the lumbar lordosis is structurally reduced as documented during standing (Inferior Spinal Angle 45.37° in young adults and 48.93° in old adults compared to 50.45° in children —not significant for OA) and a further reduction (small, but statistically significant, see Table 2) occurs at the expense of the Forward Trunk Tilt, which further increases. By considering that not only the Inferior Spinal Angle (related to lordosis) is reduced with age, but the Superior Spinal Angle (related to dorsal kyphosis) is increased in young adults (25.96°) and still more in old adults (30.34°) compared to children (16.43°), an adverse postural condition clearly appears which is associated with aging: the trunk progressively leans forward and the weight of the upper body exerts an increased flexion moment about the pelvis, which needs an increased contraction of the Erector Spinae muscles. This tendency should be counteracted as far as possible since it corresponds to increased loads on all the structural components of the spine. These results correspond to the current knowledge on the aging spine and its gradual anterior flexion [28]. In this respect, our findings support the possibility that walking is a driving factor in this evolution. Further studies could check this hypothesis.

Considering the time course of spinal length along the walking cycle (Figure 3), two peaks occur at about 45% and 95% of the stride, close to the end of each swing phase. Minimal values of spinal length occur during the double support phases. Since the spinal length increase is associated with a reduction in lumbar lordosis, and in turn, this is produced by a reduction in the anterior tilt, it appears that such a pelvic retroversion can help the swing limb to be thrown forward, increasing the step length. Of course, during the double support phase, this mechanism must switch from one limb to the other, and thus retroversion does not occur, and spinal lengthening is minimal. Interestingly, this mechanism was evident in all three groups analyzed. Regarding the range of movement, statistically significant differences were found between older adults and children concerning a few variables associated with spinal length, particularly Superior Spinal Angle and Trunk Anterior Tilt, but the increased rigidity of the column can explain this. No significant difference was observed in the range of motion of other variables that have an effect on the spinal length.

It is crucial to note the limitations of our study, particularly in the marker placement protocol, which hindered a detailed analysis of the different sagittal curves (i.e., kyphosis and lordosis) and allowed only the use of proxies that we called Superior and Inferior Spinal Angles. These limitations underscore the need to improve the methodology of trunk analysis during gait to reflect spinal physiology better, considering kyphosis and lordosis and not larger segments. Another important limitation of our study is the impossibility of drawing linear trends for variations in spine behavior during gait due to aging: the current division in age groups allows us to explore but not analyze the phenomena fully. Moreover, the older adults group did not include the elderly population, which should be studied in the future. Finally, at this early research stage, it is impossible to know if the difference between the observed variables was only statistically or clinically significant. Again, this should be further studied in the future.

## 5. Conclusions

The most significant contribution of this study is the first-time demonstration of the spine’s lengthening during gait and its change across different ages, a phenomenon previously described as a casual observation. The increase in spinal length during walking is related to a reduction in the physiological spine curve. This occurs due to the muscle contractions needed to stabilize the locomotor system. The reduction in spinal length changes with aging is associated with decreased spinal mobility and a natural tendency toward anterior trunk flexion. These results can serve as the basis for future studies in normal and pathological conditions.

## Figures and Tables

**Table 1:** Demographic and anthropometric data of the three groups. Data are shown as median (range). Statistically significant differences (*p* < 0.05): ^a^—between children and young adults; ^b^—between children and older adults, ^c^—between young and older adults, calculated using the Dunn–Sidak or Tukey–Kramer test as appropriate.

Group	Sex (Nmales/Ntot)	Age (yrs)	Height (cm)	Weight (kg)	BMI (kg/m^2^)
Children	8/15	8.0 ^a,b^ (5.0–13.0)	135.2 ^a,b^ (104.3–171.4)	31.2 ^a,b^ (20.7–65.0)	18.4 ^a,b^ (16.4–23.3)
Young adults	7/15	24.0 ^a,c^ (21.0–29.0)	168.7 ^a^ (154.5–183.6)	61.7 ^a^ (48.9–90.0)	21.9 ^a,c^ (18.6–30.6)
Older adults	8/15	60.0 ^b,c^ (54.0–66.0)	172.0 ^b^ (147.0–183.3)	74.4 ^b^ (56.0–95.6)	25.5 ^b,c^ (18.8–32.5)

**Table 2:** Average values computed during standing conditions and along the gait cycle for all the considered variables. Data are shown as median (range) for children, young adults, and older adults; *: significant difference within groups between walking and standing conditions, paired-sample *t*-test or a Wilcoxon signed rank test as appropriate; **#**: significant difference between children and young adults on the same condition, ^: significant difference between children and older adults on the same condition; significance detected with Anova or Kruskal–Wallis, with Tukey–Kramer or Dunn–Sidak post hoc comparisons as appropriate (normality tested with the Anderson–Darling test), *p* < 0.05.

		Children	Young Adults	Older Adults
Spinal Length (cm)	standing	41.06 *,#,^ (37.25–52.45)	55.32 # (47.28–61.57)	55.87 *,^ (47.81–59.70)
	walking	44.02 *,#,^ (38.95–54.01)	56.04 # (46.62–62.20)	56.50 *,^ (46.86–60.28)
Superior Spinal Angle (°)	standing	16.43 #,^ (1.83–24.56)	25.96 # (19.32–28.22)	30.34 ^ (15.72–37.25)
	walking	13.05 #,^ (5.34–24.18)	22.56 # (13.55–29.82)	31.23 ^ (18.45–36.42)
Forward Trunk Tilt (°)	standing	1.35 *,^ (0.40–3.81)	2.79 * (0.41–7.37)	3.87 *,^ (1.53–7.52)
	walking	3.61 *,#,^ (1.82–7.27)	6.07 *,# (1.57–11.24)	7.15 *,^ (3.21–9.70)
Lateral Trunk Tilt (°)	standing	0.54 (−1.18–1.27)	−0.38 (−2.53–1.27)	0.34 (−2.04–1.96)
	walking	0.001 (−0.90–1.17)	−0.14 (−1.57–0.65)	0.07 (−0.74–0.54)
Inferior Spinal Angle (°)	standing	50.45 *,# (43.53–62.41)	45.37 *,# (36.86–54.66)	48.93 * (29.07–60.20)
	walking	42.17 * (33.87–53.23)	43.55 * (37.58–48.69)	45.40 * (30.47–56.97)
Pelvic Tilt (°)	standing	12.33 *,#,^ (5.00–24.32)	6.27 # (1.40–11.16)	8.87 ^ (−10.53–13.37)
	walking	7.66 * (3.54–14.69)	5.52 (0.38–13.76)	9.55 (4.60–17.45)
Pelvic Obliquity (°)	standing	1.84 *,#,^ (−0.20–3.90)	0.18 # (−3.64–2.57)	0.43 ^ (−3.94–2.24)
	walking	0.94 * (−1.33–2.91)	0.73 (−2.76–2.39)	−0.44 (−2.16–1.80)
Pelvic Rotation (°)	standing	0.17 (−4.64–5.48)	−0.91 * (−4.53–2.95)	0.17 (−4.16–8.01)
	walking	1.80 (−1.33–5.91)	0.73 * (−1.57–4.50)	1.98 (−3.66–5.66)
Shoulder Girdle Rotation (°)	standing	−2.95 *,# (−5.22–0.58)	0.30 # (−3.14–4.01)	−1.57 (−6.35–3.39)
	walking	1.71 * (−4.21–5.31)	−0.17 (−2.47–1.84)	−0.30 (−1.61–4.84)
Shoulder Girdle Inclination (°)	standing	−0.16 (−6.04–5.14)	0.98 (−4.44–7.02)	1.77 * (−3.07–5.18)
	walking	−0.50 (−3.88–3.36)	−0.95 (−4.39–2.12)	−1.15 * (−2.71–1.48)

**Table 3:** Range of motion for all considered variables during walking. Data are shown as median (range) for children, young adults, and older adults. #: significant difference between Children and Young Adults; ^: significant difference between children and older adults, $: significant difference between young adults and older adults, tested with Anova or Kruskal–Wallis as appropriate (tested with Anderson–Darling test), Tukey–Kramer or Dunn–Sidak *p* < 0.05.

	Children	Young Adults	Older Adults
Spinal Length variation (cm)	0.65 (1.6%) (0.35–1.28)	0.95 (1.7%) $ (0.46–1.80)	0.67 (1.2%) $ (0.22–1.16)
Superior Spinal Angle (°)	2.60 ^ (1.05–5.45)	1.75 (0.98–3.13)	1.28 ^ (0.58–2.78)
Forward Trunk Tilt (°)	3.06 ^ (1.00–4.30)	2.23 (0.92–3.48)	2.10 ^ (0.70–3.33)
Lateral Trunk Tilt (°)	5.33 **#**,^ (2.397–9.19)	3.33 **#** (1.11–8.44)	3.02 ^ (1.36–5.77)
Inferior Spinal Angle (°)	2.98 (1.69–5.21)	2.63 (1.86–5.39)	2.27 (0.83–3.74)
Pelvic Tilt (°)	2.83 (1.09–4.57)	2.67 (1.01–3.77)	2.25 (1.11–3.09)
Pelvic Obliquity (°)	8.55 ^ (5.35 –11.84)	7.67 (4.95–14.21)	6.72 ^ (2.68–10.24)
Pelvic Rotation (°)	15.48 ^ (7.39–26.71)	11.54 $ (8.24–16.88)	7.74 ^,$ (1.82–16.79)
Shoulder Girdle Rotation (°)	11.91 (8.50–20.60)	13.54 (7.57–17.10)	10.84 (5.97–16.72)
Shoulder Girdle Inclination (°)	16.49 (10.04–30.73)	16.86 (10.67–22.69)	11.55 ^ (5.44–20.28)

## Data Availability

The data presented in this study are available on request from the corresponding author.

## References

[1] Smania N., Picelli A., Romano M., Negrini S. (2008). Neurophysiological Basis of Rehabilitation of Adolescent Idiopathic Scoliosis. Disabil. Rehabil..

[2] Paramento M., Passarotto E., Maccarone M.C., Agostini M., Contessa P., Rubega M., Formaggio E., Masiero S. (2024). Neurophysiological, Balance and Motion Evidence in Adolescent Idiopathic Scoliosis: A Systematic Review. PLoS ONE.

[3] Cury A.C., Pinto R.Z., Madaleno F.O., Resende R.A. (2021). Do Older Adults Present Altered Pelvic and Trunk Movement Pattern during Gait? A Systematic Review with Meta-Analysis and GRADE Recommendations. Braz. J. Phys. Ther..

[4] Loughenbury P.R., Tsirikos A.I., Gummerson N.W. (2016). Spinal Biomechanics—Biomechanical Considerations of Spinal Stability in the Context of Spinal Injury. Orthop. Trauma.

[5] Gracovetsky S. (1997). Linking the Spinal Engine with the Legs: A Theory of Human Gait. Movement, Stability and Low Back Pain.

[6] Frigo C., Pavan E. (2004). ESMAC Abstracts 2004. S2.4: Are We Taller during Walking than during Standing?. Gait Posture.

[7] Granacher U., Lacroix A., Muehlbauer T., Roettger K., Gollhofer A. (2013). Effects of Core Instability Strength Training on Trunk Muscle Strength, Spinal Mobility, Dynamic Balance and Functional Mobility in Older Adults. Gerontology.

[8] Shahtahmassebi B., Hebert J.J., Hecimovich M.D., Fairchild T.J. (2017). Associations Between Trunk Muscle Morphology, Strength and Function in Older Adults. Sci. Rep..

[9] Frigo C., Carabalona R., Dalla Mura M., Negrini S. (2003). The Upper Body Segmental Movements during Walking by Young Females. Clin. Biomech..

[10] Crosbie J., Vachalathitib R., Smith R. (1997). Age, Gender and Speed Effects on Spinal Kinematics during Walking. Gait Posture.

[11] Schmid S., Bruhin B., Ignasiak D., Romkes J., Taylor W.R., Ferguson S.J., Brunner R., Lorenzetti S. (2017). Spinal Kinematics during Gait in Healthy Individuals across Different Age Groups. Hum. Mov. Sci..

[12] Yang J.H., Suh S.W., Sung P.S., Park W.H. (2013). Asymmetrical Gait in Adolescents with Idiopathic Scoliosis. Eur. Spine J..

[13] Nishi Y., Shigetoh H., Fujii R., Osumi M., Morioka S. (2021). Changes in Trunk Variability and Stability of Gait in Patients with Chronic Low Back Pain: Impact of Laboratory versus Daily-Living Environments. J. Pain Res..

[14] Rahman S., Griffin H.J., Quinn N.P., Jahanshahi M. (2008). Quality of Life in Parkinson’s Disease: The Relative Importance of the Symptoms. Mov. Disord..

[15] Wang C., Li X., Guo Y., Du W., Guo H., Chen W. (2022). The Kinematic and Kinetic Responses of the Trunk and Lower Extremity Joints during Walking with and without the Spinal Orthosis. Int. J. Environ. Res. Public Health.

[16] World Medical Association Declaration of Helsinki (2013). Ethical Principles for Medical Research Involving Human Subjects. JAMA.

[17] Ahmed Shalaby S. (2015). Morphometric Study of the Normal Egyptian Coccyx from (Age 1–40 Year). Int. J. Clin. Dev. Anat..

[18] Woon J.T.K., Perumal V., Maigne J.Y., Stringer M.D. (2013). CT Morphology and Morphometry of the Normal Adult Coccyx. Eur. Spine J..

[19] Tsuruta A., Tashiro J., Ishii T., Oka Y., Suzuki A., Kondo H., Yamaguchi S. (2017). Prediction of Anastomotic Leakage After Laparoscopic Low Anterior Resection in Male Rectal Cancer by Pelvic Measurement in Magnetic Resonance Imaging. Surg. Laparosc. Endosc. Percutan. Tech..

[20] Palmisano C., Farinelli V., Camuncoli F., Favata A., Pezzoli G., Frigo C.A., Isaias I.U. (2024). Dynamic Evaluation of Spine Kinematics in Individuals with Parkinson’s Disease and Freezing of Gait. Gait Posture.

[21] Kirtley C. (2006). Clinical Gait Analysis.

[22] Penny K.I. (1996). Appropriate Critical Values When Testing for a Single Multivariate Outlier by Using the Mahalanobis Distance. Appl. Stat..

[23] Dreischarf M., Albiol L., Rohlmann A., Pries E., Bashkuev M., Zander T., Duda G., Druschel C., Strube P., Putzier M. (2014). Age-Related Loss of Lumbar Spinal Lordosis and Mobility—A Study of 323 Asymptomatic Volunteers. PLoS ONE.

[24] Granacher U., Gollhofer A., Hortobágyi T., Kressig R.W., Muehlbauer T. (2013). The Importance of Trunk Muscle Strength for Balance, Functional Performance, and Fall Prevention in Seniors: A Systematic Review. Sports Med..

[25] Lamoth C.J.C., Meijer O.G., Wuisman P.I.J.M., Van Dieën J.H., Levin M.F., Beek P.J. (2002). Pelvis-Thorax Coordination in the Transverse Plane During Walking in Persons with Nonspecific Low Back Pain. Spine.

[26] Perry J., Burnfield J. (2010). Gait Analysis: Normal and Pathological Function.

[27] Ceccato J.C., de Sèze M., Azevedo C., Cazalets J.R. (2009). Comparison of Trunk Activity during Gait Initiation and Walking in Humans. PLoS ONE.

[28] Barrey C., Roussouly P., Le Huec J.-C., D’Acunzi G., Perrin G. (2013). Compensatory Mechanisms Contributing to Keep the Sagittal Balance of the Spine. Eur. Spine J..

